# Exercise Modifies the Gut Microbiota with Positive Health Effects

**DOI:** 10.1155/2017/3831972

**Published:** 2017-03-05

**Authors:** Vincenzo Monda, Ines Villano, Antonietta Messina, Anna Valenzano, Teresa Esposito, Fiorenzo Moscatelli, Andrea Viggiano, Giuseppe Cibelli, Sergio Chieffi, Marcellino Monda, Giovanni Messina

**Affiliations:** ^1^Department of Experimental Medicine, Section of Human Physiology and Unit of Dietetic and Sport Medicine, Second University of Naples, Naples, Italy; ^2^Department of Clinical and Experimental Medicine University of Foggia, Foggia, Italy; ^3^Department of Medicine, Surgery, and Dentistry “Scuola Medica Salernitana”, University of Salerno, Salerno, Italy

## Abstract

The human gastrointestinal tract (GIT) is inhabited by a wide cluster of microorganisms that play protective, structural, and metabolic functions for the intestinal mucosa. Gut microbiota is involved in the barrier functions and in the maintenance of its homeostasis. It provides nutrients, participates in the signaling network, regulates the epithelial development, and affects the immune system. Considering the microbiota ability to respond to homeostatic and physiological changes, some researchers proposed that it can be seen as an endocrine organ. Evidence suggests that different factors can determine changes in the gut microbiota. These changes can be both quantitative and qualitative resulting in variations of the composition and metabolic activity of the gut microbiota which, in turn, can affect health and different disease processes. Recent studies suggest that exercise can enhance the number of beneficial microbial species, enrich the microflora diversity, and improve the development of commensal bacteria. All these effects are beneficial for the host, improving its health status. In this paper, we intend to shed some light over the recent knowledge of the role played by exercise as an environmental factor in determining changes in microbial composition and how these effects could provide benefits to health and disease prevention.

## 1. Introduction

Intestinal microbiome has protective, structural, and metabolic functions in the intestinal mucosa [1, 2]. A lot of current knowledge about these functions is due to the use of germ-free (GF) animals, in which postnatal colonization of the gastrointestinal tract was prevented through surgical delivery instead of natural childbirth [3]. Comparing these animals with normal controls, several studies have demonstrated that GF animals present a reduction of the intestinal surface area, thinner villous, and smaller Peyer's Patches [1]. Several researchers focused on gut microbes to better understand their functions, characteristics, and impact on human health. The metabolic activity of the gut microflora is comparable to that of an organ inside another organ, being able to influence the mucosal homeostasis and immune responses [2]. Furthermore, gut microflora provides nutrients, regulates the epithelial development, and affects the immune system [4]. Consequently, it appears as an essential organ and knowledge about it could help in understanding the factors that influence human health and disease processes, such as inflammation, infections, and tumors [4]. In light of this, humans may be considered as a superorganism in which microbes and human attributes determine their metabolism [1]. Among healthy subjects, there is a high interindividual variability in the composition of the gut microflora and an enriched microbial diversity is associated with improvement in health status and variations in immune system. These observations suggest the presence of different host-microbiota correlations [4, 5]. Yet, the gut ecosystem development and its stability can be influenced by an existing dynamic balance between intrinsic and extrinsic factors such as host physiology, lifestyle, exercise, and diet, which in turn can impact health [6]. For example, decreased microbiota diversity and a higher ratio Firmicutes:Bacteroidetes are associated with obesity type II diabetes and altered blood glucose [7, 8]. On the other hand, exercise and high fiber diet, such as fruits, vegetables, legumes, and whole-wheat grain products, increase the microbic diversity [9, 10]. Recent studies suggest that an increase in exercise can enhance the number of beneficial microbial species and that the microbiota is responsive to the homeostatic and physiological variations due to exercise [5, 11]. In this paper, we review the recent knowledge of the role played by exercise as an environmental factor in determining changes in the microbial composition and how these effects could provide benefits to health and disease prevention.

## 2. The Gut Microbiota Composition and Development

The human gastrointestinal tract (GIT) is inhabited by 1013-1014 microorganisms and their genome, the “microbiome,” contains a gene set which is about 150 times greater than that of human genome [12, 13]. This microbiome derives from about 1,000–1,150 bacterial species, mostly anaerobes, and it is primarily made up of two bacterial phyla, Bacteroidetes and Firmicutes [4, 13]. There is great variability in the number, type, and function of microorganisms along the entire GIT but most are located in the large bowel where they ferment nondigestible food components allowing us to obtain otherwise inaccessible nutrients [14, 15]. Microbiome development starts in early life. In fact, during fetal life, the GIT is sterile due to the sterile uterine environment [6]. After birth, the infant gut is exposed to complex surrounding environment and maternal microflora which begin to colonize the GIT, showing an initial microbiome with a maternal signature [6, 16]. Several intrinsic and extrinsic factors influence the development and variation of bacteria in infants (such as the passage from liquid to solid feed), so that the GIT is colonized by different microorganisms. By 1 year of age the microbiome presents an adult-like profile with a dense microbial population [17, 18]. Moreover, also genetic, epigenetic and environmental factors, like country of origin and antibiotics, and life events (including puberty, ovarian cycle, pregnancy, and menopause) affect the microbial population development and its activity [3, 19]. This population, once established, presents a high interindividual variability [20]. Furthermore, the gut microbe populations change in old age showing a significant decrease in Bacteroidetes and an increase in Firmicutes, but the reason for this is not yet clear [19].

## 3. Functions of the Intestinal Microbiome

The gut microbiota plays various important functions for the host health. The gut microbiota is essential for the motility of the gastrointestinal tract, facilitating peristalsis [21], and it is involved in the fortification of the barrier and in the maintenance of its homeostasis. This has been proven by the fact that the recognition of commensal bacteria by toll-like receptors (TLRs) is necessary to stimulate the epithelial cell proliferation, protecting the epithelial surface against gut injury [22]. Furthermore, Paneth cells, which are secretory cells of the small intestine epithelium, perceive enteric bacteria through TRLs activation and trigger the expression of various antimicrobial factors. This allows exerting control over intestinal barrier penetration by pathogenic bacteria [23]. The microbiota is also related to the development of the gut associated lymphoid tissue (GALT), the host immune system stimulating IgA secretion and inhibiting colonization of the GIT by pathogens [21, 24, 25]. In addition, protective functions are performed by the microbiota through competition with pathogens for nutrients and receptors and the production of antimicrobial molecules to avoid colonization by pathogens [26]. Through ligands from commensal bacteria (as lipopolysaccharide, LPS), the gut microbiota influences the mucosal immune system development and function [22]. The innate immune system can also recognize potentially pathogenic microbes through TLRs identification of particular molecules called pathogen associated molecular patterns (PAMP) [27]. This leads to an increase in cytokine levels and T-cell activation which are necessary for appropriate immune responses to pathogens [2, 21]. The microbiota has also important effects on metabolic functions. It can ferment nondigestible dietary residues producing short-chain fatty acids (SCFAs, such as n-butyrate, acetate, and propionate) which, in turn, can modulate the host energy balance increasing the nutrients availability [28]. SCFAs, secreted into the gut lumen, exceed the epithelial barrier and are released into the bloodstream. In this way they reach different organs and may be used as substrates for energy metabolism; hepatocyte cells, in particular, use propionate for gluconeogenesis [28]. SCFAs are involved in the gut-brain axis, stimulating the release of peptide YY (PYY) and 5-hydroxytryptamine (5-HT). They also act as signaling molecules to regulate immune and inflammatory responses [29, 30]. For instance, n-butyrate regulates neutrophil function and migration, increases the expression of tight junction proteins in colon epithelia, reduces mucosal permeability, and inhibits inflammatory cytokines [19]. Beside producing SCFAs, bacterial species of the intestinal microbiota synthesize glycan, amino acids, and vitamins (e.g., K, B12, Biotin, Folate, and Thiamine), thus participating in the host metabolism [1, 12, 19, 31].

## 4. Microbiota and Diseases

The gut microbiota is essential to maintain homeostasis and normal gut physiology [1]. Several diseases have been associated with an altered composition of the microbiota, such as obesity, coronary heart disease, diabetes, and inflammatory bowel disease [32–37]. These diseases have a multifactorial origin, comprising environmental and genetic factors. In recent years, the contribution of the microbiota is considered an important environmental factor [38]. Ley and coworkers (2005) [32] have shown that genetically obese mice (*ob/ob *mice) exhibit a strong reduction of Bacteroidetes and an increase of Firmicutes. In humans, lower levels of Bacteroides and higher levels of Firmicutes are also present in the fecal microbiota of obese individuals when compared with lean controls. Interestingly, the ratio between the two phyla can be reversed by a caloric restricted diet [20]. Alterations in microbiota composition are also associated with inflammatory bowel disease (IBD), a gastrointestinal disorder that encloses both ulcerative colitis (UC) and Crohn's disease (CD) [39, 40]. These alterations are characterized by a reduction of Firmicutes and Bacteroidetes and increase in Proteobacteria. However, it is unclear if this contributes to IBD or is a consequence of the inflammatory state related to IBD [37, 41]. Furthermore, in the IBD pathogenesis, psychological stress has been recognized as a factor that can influence the microflora and worsen the physical state [42]. Another gastrointestinal disorder, in which the microbiota plays an important role, is the irritable bowel syndrome (IBS). The IBS is a stress-related brain-gut axis disorder characterized by abdominal pain or discomfort and alteration in intestinal habit [43]. Studies on postinfectious IBS (IBS development following a bacterial gastroenteritis) supported a link between the dysfunctions in the microflora and mucosal inflammation [44]. In IBS patients, the microflora shows a doubled increased ratio of the Firmicutes to Bacteroidetes, a reduction in the number of Bacteroidetes, and an increase in numbers of* Dorea, Ruminococcus*, and* Clostridium *spp. compared with healthy controls [45]. Even in this disorder, it is unclear whether these microflora alterations are a cause or an effect of the pathophysiology [44].

## 5. Gut Microbiota, Exercise, and Disease

In spite of the great interindividual variation in the GIT microbial composition, its reduction or alteration is associated with negative health effects. On the other hand, an increase in the diversity of intestinal population improves metabolic and immunological functions [4, 46]. An increasing body of evidence suggests that gut microbiota can be modulated by different factors, such as infection, disease, diet, antibiotics, and exercise, and, in turn, these modulations can affect some diseases [1, 6]. Interestingly, exercise can determine changes in the gut microbial composition playing a positive role in energy homeostasis and regulation [5, 11].

### 5.1. Exercise and Gut Physiology

Low intensity exercise can influence the GIT reducing the transient stool time and thus the contact time between the pathogens and the gastrointestinal mucus layer [5]. As a consequence, it seems that exercise has protective effects, reducing the risk of colon cancer, diverticulosis, and inflammatory bowel disease [47]. In addition, even in the presence of high fat diet, exercise may reduce inflammatory infiltrate and protect the morphology and the integrity of the intestine [48]. High fat diet, accompanied with sedentary behavior, leads to increased villi width due to plasmacytoid and lymphocytic infiltrates. Exercise prevented these morphological changes by reducing cyclooxygenase 2 (Cox-2) expression in both proximal and distal gut. Conversely, it appears that endurance exercise determines a variation in the GIT due to the reduction of the splanchnic blood flow, as much as 80% of basal levels, resulting in toxicity effects [47, 49]. This reduction depends on the increase of arterial resistance in the splanchnic vascular bed, secondary to augmentation of sympathetic nervous system input [47]. Prolonged exercise also determines an increase of intestinal permeability, compromising gut-barrier function and resulting in bacterial translocation from the colon [47, 50].

### 5.2. Voluntary Exercise and Gut Microflora

The earliest evidence about the effects of voluntary exercise on the gut microbiota is derived from observations of Matsumoto and colleagues [51]. The authors [51] reported that, in rats, voluntary running exercise determined a variation in microbiota composition, an increase of n-butyrate concentration, and an increase in the cecum diameter. Since n-butyrate protects against colon cancer and IBD affecting cellular NF-B activation [52], Matsumoto et al. [51] proposed that the increase in n-butyrate is involved in the reduction of the colon diseases risk associated with exercise. In addition, Evans et al. [53] have demonstrated that, in obese-induced mice through high fat feeding, exercise can prevent obesity and induces changes in the percentage of major bacterial phyla. Furthermore, Evans et al. [53] found that the total distance run was inversely correlated with the Bacteroidetes-Firmicutes ratios. The authors [53] suggested that exercise plays an important role in prevention of diet-induced obesity producing a microbial composition similar to lean mice [53]. Similar results were found by Campbell et al. [48]. They [48] showed that exercise manifested a unique microbiome independent of diet. Moreover, Campbell et al. [48] have suggested that in exercised mice there are bacteria related to* Faecalibacterium prausnitzii* which may protect the digestive tract by producing butyrate and lowering the oxygen tension in the lumen by a flavin/thiol electron shuttle [48]. On the other hand, the association between food restriction and exercise seems to determine a decrease of beneficial bacteria and an increase of bacteria that cause gut mucosal barrier disorders [54]. Moreover, serum leptin levels show a positive correlation with the quantity of* Bifidobacterium *and* Lactobacillus *and a negative correlation with the quantity of* Bacteroides *and* Prevotella*. Serum ghrelin levels show an inverse correlation with these bacteria [54]. These series of evidence demonstrate that nutritional status and exercise influence gut microbiota and that the gut microbiota is associated with appetite; regulating hormones have investigated whether, in rats, there were differences in the microbial composition when exercise started in the juvenile period or in adulthood. The authors observed that when exercise started in juvenile period it modified various phyla with an increase of Bacteroidetes and a decrease of Firmicutes [11]. Furthermore, juveniles exercise, compared with adult exercise, modified more genera and led to an increase in lean body mass [11]. These data suggest that early life exercise can influence the gut microbiota composition stimulating the development of bacteria able to determine adaptive changes in host metabolism [11]. Furthermore, exercise initiated in early life may favor optimal development of brain function, promoting health-enhancing microbial species [55]. Using GF mice models, recent studies suggested that the gut microbiota may alter brain function [56–58]. For example, in rats,* Lactobacillus rhamnosus* can reduce anxiety and depressive-like behavior, attenuate hypothalamic-pituitary-adrenal axis activation following a stress challenge, and produce changes in GABA receptor expression via the vagus nerve [59]. Evidence suggests that different metabolites and signaling molecules produced by gut microorganisms (as SCFAs) can activate vagal afferents receptors of the enteric nervous system [59]. These signals are propagated by the nucleus of the solitary tract to various projection regions, such as limbic structures important for mood and behavior [55]. Therefore, especially during juvenile period, exercise and gut microbiota represent important factors to promote both brain and metabolic development [11, 55].

### 5.3. Controlled Exercise and Gut Microbiota

Although exercise-altered microbiota could be an approach for the treatment of diseases associated with alterations of the intestinal microflora, very few studies have investigated the beneficial effects of exercise on the microflora composition in relation to disease. Among these studies, Cook et al. [60] have highlighted the effects of habitual exercise on gut health and disease. They [60] stressed that exercise played an anti-inflammatory action in the gut, although in mice, different forms of exercise training induced distinct effects on the gut microbiome during an inflammatory insult. Specifically, forced and voluntary exercise differentially altered the microbiome in both the cecum and feces of mice, resulting in different microbial taxonomy [60]. These microbial changes may be related to gut immune function and microbiota-immune interactions and they may also be involved in the pathogenesis of IBD, nutrient absorption, immune function, and host physiology [61]. Petriz et al. [62] examined the effect of controlled exercise training on the gut microbiome of obese and hypertensive rats. They found that nonobese and hypertensive rats showed a different composition of the intestinal microflora compared with the obese rats. Furthermore, exercise led to an improvement in the composition and diversity of gut bacteria. Petriz et al. suggested that the exercise may be a therapeutic approach for obesity and/or hypertension through the modulation of gut microbiota [62]. Other studies in rats demonstrated that high fat diet (HFD) determines obesity which, in turn, decreased plasticity and led to anxiety and cognitive problems [63–66]. On the other hand, exercise can improve the cognitive decline associated with HFD [63–65]. Moreover, some studies have demonstrated that diet induces changes in bacteria diversity which, in turn, can influence anxiety, memory, and learning [67, 68]. Based on these observations, Kang et al. [69] investigated the effects of HFD and controlled the effects of exercise on the gut microbiome. The authors observed that HFD determined anxiety phenotypes that were not rescued by exercise, while exercise increased cognitive abilities without being influenced by the HFD. Furthermore, they found that exercise determined changes in the gut microbiome and the levels of some specific bacteria (such as, Lachnospiraceae and Ruminococcaceae) were directly proportional to anxiety or cognition. Kang et al. [69] proposed that diet and exercise influence the behavior and the gut microbiome even if in unrelated ways. Exercise determines also an increase in lactic acid bacteria (LAB). LAB are associated with the mucosal surface of the GIT and produce lactic acid that can modulate mucosal immunity and exclusion of pathogens [64, 70]. Also the levels of* B. coccoides *and* E. rectal *are increased with the exercise and, in the gut, they convert the lactate derived from LAB into butyrate which, in turn, plays an important role in the mucin synthesis and gut epithelium protection [54, 59].

### 5.4. Exercise and Human Gut Microbiota

In humans, a major study conducted on elite rugby players demonstrated that exercise enriched the diversity of gut microflora and positively correlated with protein intake and creatine kinase levels [10]. In particular, there was a greater diversity among the Firmicutes phylum (such as* Faecalibacterium prausnitzii*) that helped to maintain a healthier intestinal environment [10]. These results indicated that both diet and exercise determined the microbial biodiversity of the gut. In support of this, Estaki et al. [71] analyzed the fecal microbiota of individuals with different fitness levels and comparable diets. As indicator of physical fitness, they used peak oxygen uptake, the gold standard of cardiorespiratory fitness (CRF). The results demonstrated that, regardless of diet, CRF was correlated with increased gut microbial diversity. Furthermore fit individuals showed a microbiome enriched in butyrate-producing taxa, such as Clostridiales,* Roseburia*, Lachnospiraceae, and Erysipelotrichaceae, resulting in increased butyrate production, an indicator of gut health [71]. Estaki et al. [71] proposed that exercise could be used as a therapeutic support in the treatment of dysbiosis-associated diseases. Increased diversity is associated with increased health also in the elderly, while, reduction of biodiversity is linked to different conditions such as obesity-associated inflammatory characteristics and gastrointestinal diseases (as IBD and IBS) [32, 40, 43, 72, 73]. Then the increase of microbial biodiversity related to the exercise could have beneficial effects on the pathogenesis of these conditions. Furthermore, since athletes show lower inflammatory and improved metabolic markers relative to controls, and the exercise is associated with reduced morbidity due to lower chronic inflammation, it is possible to hypothesize that age-appropriate exercise and diet could help to decrease inflammation and age-related pathologies [10, 71, 74–76]. Moreover, compared with subjects with high BMI, subjects with low BMI and athletes show higher* Akkermansia muciniphila *levels in their microflora [10]. These bacteria are mucin-degrading bacteria which reside in the mucus layer and they are inversely correlated with BMI, obesity, and metabolic disorders probably because they improve barrier function [77]. Juneau et al. [78] suggested that the combination of high-intensity interval training (HIIT) and high-quality diet could prevent cardiovascular (CV) disease development. Other studies, instead, investigated the effects of exercise on the microflora of obese subjects. In particular, obesity, through inflammation, insulin resistance, and visceral adiposity, is also considered a major cause of several sleep disorders, such as obstructive sleep apnea (OSA) sleepiness, and the associated cardiovascular comorbidities [79]. In subjects with obesity-related sleep disorders, some researchers investigated the effects of exercise and diet and observed that these factors determined an improvement of sleep quality and changes in the gut microbiota composition [80]. Alterations in microbiome were also present in subjects with myalgic encephalomyelitis/chronic fatigue syndrome (ME/CFS), a disease characterized by intense and debilitating fatigue not due to physical activity and associated with neuroinflammatory and oxidative processes [81–83]. Patients with ME/CFS showed a worsening of symptoms following exercise associated with intestinal dysbiosis. This could be due to increased intestinal permeability and increased bacterial translocation from the intestine into the bloodstream, resulting in further inflammation which, in turn, contributed to increase ME/CFS symptoms (such as pain, fatigue, and mood) [83]. In these patients, the characterization of the gut microbiome demonstrated significant alterations compared with healthy controls with an increase of Firmicutes, particularly of* Clostridium* spp., in blood samples after exercise [81–84]. In light of this, it was suggested that the recognition of changes in the intestinal microflora and bacterial translocation into the bloodstream in response to exercise could be a method to evaluate the effectiveness of treatments of these patients.

## 6. Exercise, Probiotic Supplementation, and Gut Microbiota

Some studies evaluated how the use of probiotics could modify the microbiota composition. Probiotics are food supplements containing live microorganisms, generally lactic acid bacteria, which give beneficial effects for the host [84]. Chen et al. [85] examined the effects of six weeks of supplementation with probiotics,* Lactobacillus plantarum* TWK10 (LP10), on exercise performance, physical fatigue, and gut microbial profile in mice. Their results show that LP10 supplementation increased muscle mass and grip strength in a dose-dependent way and enhanced energy harvesting and exercise performance [85]. It was possible that* Lactobacillus *spp. influenced exercise performance by producing lactic acid, which, in turn, could be used by lactate-utilizing bacteria to produce butyrate [86]. Along this pathway, there was formation of adenosine triphosphate (ATP). Thus, probiotic supplementation could play important roles in energy production during exercise [86]. Furthermore, Chen and coworkers [85] showed that LP10 supplementation had antifatigue effects by decreasing levels of serum lactate, ammonia, and creatine kinase (biochemical indicators of exercise-induced muscle fatigue) and enhanced exercise performance in mice. This might be related to the reduction of inflammation induced by LP10 which determined an improvement of skeletal muscle atrophy markers [85]. These findings support the view that gut microbiota had health-promotion, performance-improvement, and antifatigue effects on the host during exercise in terms of energy balance and body composition.

## 7. Conclusion

Collectively, the available data strongly support that, in addition to other well-known internal and external factors, exercise appears to be an environmental factor that can determine changes in the qualitative and quantitative gut microbial composition with possible benefits for the host. In fact, stable and enriched microflora diversity is indispensable to the homeostasis and normal gut physiology contributing also to suitable signaling along the brain-gut axis and to the healthy status of the individual. Exercise is able to enrich the microflora diversity; to improve the Bacteroidetes-Firmicutes ratio which could potentially contribute to reducing weight, obesity-associated pathologies, and gastrointestinal disorders; to stimulate the proliferation of bacteria which can modulate mucosal immunity and improve barrier functions, resulting in reduction in the incidence of obesity and metabolic diseases; and to stimulate bacteria capable of producing substances that protect against gastrointestinal disorders and colon cancer (such as, SCFAs). Therefore the exercise can be used as a treatment to maintain the balance of the microflora or to rebalance his eventual dysbiosis, thus obtaining an improvement of the health status. Nevertheless further studies are needed to fully understand the mechanisms that determine changes in the composition and functions of the microflora caused by exercise and all their related effects. In addition exercise-altered microbiota could be used to look for new approaches in the treatment of metabolic and inflammatory diseases in which it is well known that the microbiota plays an important role.

## References

[1] Grenham S., Clarke G., Cryan J. F., Dinan T. G. (2011). Brain-gut-microbe communication in health and disease. *Frontiers in Physiology*.

[2] O'Hara A. M., Shanahan F. (2006). The gut flora as a forgotten organ. *EMBO Reports*.

[3] Adlerberth I., Wold A. E. (2009). Establishment of the gut microbiota in Western infants. *Acta Paediatrica, International Journal of Paediatrics*.

[4] Eckburg P. B., Bik E. M., Bernstein C. N. (2005). Microbiology: diversity of the human intestinal microbial flora. *Science*.

[5] Bermon S., Petriz B., Kajeniene A., Prestes J., Castell L., Franco O. L. (2015). The microbiota: an exercise immunology perspective. *Exercise Immunology Review*.

[6] Mackie R. I., Sghir A., Gaskins H. R. (1999). Developmental microbial ecology of the neonatal gastrointestinal tract. *The American Journal of Clinical Nutrition*.

[7] Payne A. N., Chassard C., Lacroix C. (2012). Gut microbial adaptation to dietary consumption of fructose, artificial sweeteners and sugar alcohols: implications for host-microbe interactions contributing to obesity. *Obesity Reviews*.

[8] Remely M., Aumueller E., Jahn D., Hippe B., Brath H., Haslberger A. G. (2014). Microbiota and epigenetic regulation of inflammatory mediators in type 2 diabetes and obesity. *Beneficial Microbes*.

[9] Flint H. J., Scott K. P., Louis P., Duncan S. H. (2012). The role of the gut microbiota in nutrition and health. *Nature Reviews Gastroenterology & Hepatology*.

[10] Clarke S. F., Murphy E. F., O'Sullivan O. (2014). Exercise and associated dietary extremes impact on gut microbial diversity. *Gut*.

[11] Mika A., Van Treuren W., González A., Herrera J. J., Knight R., Fleshner M. (2015). Exercise is more effective at altering gut microbial composition and producing stable changes in lean mass in juvenile versus adult male F344 rats. *PLoS ONE*.

[12] Gill S. R., Pop M., DeBoy R. T. (2006). Metagenomic analysis of the human distal gut microbiome. *Science*.

[13] Qin J., Li R., Raes J. (2010). A human gut microbial gene catalogue established by metagenomic sequencing. *Nature*.

[14] Conlon M. A., Bird A. R. (2015). The impact of diet and lifestyle on gut microbiota and human health. *Nutrients*.

[15] Bäckhed F., Ley R. E., Sonnenburg J. L., Peterson D. A., Gordon J. I. (2005). Host-bacterial mutualism in the human intestine. *Science*.

[16] Mändar R., Mikelsaar M. (1996). Transmission of mother’s microflora to the newborn at birth. *Neonatology*.

[17] Palmer C., Bik E. M., DiGiulio D. B., Relman D. A., Brown P. O., Ruan Y. (2007). Development of the human infant intestinal microbiota. *PLOS Biology*.

[18] Tannock G. W. (2007). What immunologists should know about bacterial communities of the human bowel. *Seminars in Immunology*.

[19] Nicholson J. K., Holmes E., Kinross J. (2012). Host-gut microbiota metabolic interactions. *Science*.

[20] Ley R. E., Peterson D. A., Gordon J. I. (2006). Ecological and evolutionary forces shaping microbial diversity in the human intestine. *Cell*.

[21] Berg R. D. (1996). The indigenous gastrointestinal microflora. *Trends in Microbiology*.

[22] Rakoff-Nahoum S., Paglino J., Eslami-Varzaneh F., Edberg S., Medzhitov R. (2004). Recognition of commensal microflora by toll-like receptors is required for intestinal homeostasis. *Cell*.

[23] Vaishnava S., Behrendt C. L., Ismail A. S., Eckmann L., Hooper L. V. (2008). Paneth cells directly sense gut commensals and maintain homeostasis at the intestinal host-microbial interface. *Proceedings of the National Academy of Sciences*.

[24] Mayer L. (2003). Mucosal immunity. *Pediatrics*.

[25] Messina G., Dalia C., Tafuri D. (2014). Orexin-A controls sympathetic activity and eating behavior. *Frontiers in Psychology*.

[26] Sekirov I., Russell S. L., Caetano M Antunes L., Finlay B. B. (2010). Gut microbiota in health and disease. *Physiological Reviews*.

[27] Akira S., Hemmi H. (2003). Recognition of pathogen-associated molecular patterns by TLR family. *Immunology Letters*.

[28] Samuel B. S., Shaito A., Motoike T. (2008). Effects of the gut microbiota on host adiposity are modulated by the short-chain fatty-acid binding G protein-coupled receptor, Gpr41. *Proceedings of the National Academy of Sciences of the United States of America*.

[29] Evans J. M., Morris L. S., Marchesi J. R. (2013). The gut microbiome: the role of a virtual organ in the endocrinology of the host. *Journal of Endocrinology*.

[30] Maslowski K. M., Vieira A. T., Ng A. (2009). Regulation of inflammatory responses by gut microbiota and chemoattractant receptor GPR43. *Nature*.

[31] Viggiano A., Nicodemo U., Viggiano E. (2010). Mastication overload causes an increase in O_2_^−^ production into the subnucleus oralis of the spinal trigeminal nucleus. *Neuroscience*.

[32] Ley R. E., Bäckhed F., Turnbaugh P., Lozupone C. A., Knight R. D., Gordon J. I. (2005). Obesity alters gut microbial ecology. *Proceedings of the National Academy of Sciences of the United States of America*.

[33] Fava F., Lovegrove J. A., Gitau R., Jackson K. G., Tuohy K. M. (2006). The gut microbiota and lipid metabolism: implications for human health and coronary heart disease. *Current Medicinal Chemistry*.

[34] Wen L., Ley R. E., Volchkov P. Y. (2008). Innate immunity and intestinal microbiota in the development of Type 1 diabetes. *Nature*.

[35] Viggiano A., Vicidomini C., Monda M. (2009). Fast and low-cost analysis of heart rate variability reveals vegetative alterations in noncomplicated diabetic patients. *Journal of Diabetes and its Complications*.

[36] Di Bernardo G., Messina G., Capasso S. (2014). Sera of overweight people promote in vitro adipocyte differentiation of bone marrow stromal cells. *Stem Cell Research & Therapy*.

[37] Frank D. N., St Amand A. L., Feldman R. A., Boedeker E. C., Harpaz N., Pace N. R. (2007). Molecular-phylogenetic characterization of microbial community imbalances in human inflammatory bowel diseases. *Proceedings of the National Academy of Sciences of the United States of America*.

[38] Bäckhed F., Ding H., Wang T. (2004). The gut microbiota as an environmental factor that regulates fat storage. *Proceedings of the National Academy of Sciences of the United States of America*.

[39] Lichtenstein G. R. (2000). Chemokines and cytokines in inflammatory bowel disease and their application to disease treatment. *Current Opinion in Gastroenterology*.

[40] Shanahan F. (2004). Probiotics in inflammatory bowel disease—therapeutic rationale and role. *Advanced Drug Delivery Reviews*.

[41] Macfarlane G. T., Blackett K. L., Nakayama T., Steed H., Macfarlane S. (2009). The gut microbiota in inflammatory bowel disease. *Current Pharmaceutical Design*.

[42] Mawdsley J. E., Rampton D. S. (2007). The Role of Psychological Stress in Inflammatory Bowel Disease. *Neuroimmunomodulation*.

[43] Thompson W. G., Longstreth G. F., Drossman D. A., Heaton K. W., Irvine E. J., Müller-Lissner S. A. (1999). Functional bowel disorders and functional abdominal pain. *Gut*.

[44] Quigley E. M. (2009). Review: do patients with functional gastrointestinal disorders have an altered gut flora?. *Therapeutic Advances in Gastroenterology*.

[45] Rajilić-Stojanović M., Biagi E., Heilig H. G. H. J. (2011). Global and deep molecular analysis of microbiota signatures in fecal samples from patients with irritable bowel syndrome. *Gastroenterology*.

[46] Cryan J. F., O'Mahony S. M. (2011). The microbiome-gut-brain axis: from bowel to behavior. *Neurogastroenterology and Motility*.

[47] Peters H. P. F., De Vries W. R., Vanberge-Henegouwen G. P., Akkermans L. M. A. (2001). Potential benefits and hazards of physical activity and exercise on the gastrointestinal tract. *Gut*.

[48] Campbell S. C., Wisniewski P. J., Noji M. (2016). The effect of diet and exercise on intestinal integrity and microbial diversity in mice. *PLoS ONE*.

[49] Rehrer N. J., Smets A., Reynaert H., Goes E., De Meirleir K. (2001). Effect of exercise on portal vein blood flow in man. *Medicine and Science in Sports and Exercise*.

[50] Gisolfi C. V. (2000). Is the GI system built for exercise?. *Physiology*.

[51] Matsumoto M., Inoue R., Tsukahara T. (2008). Voluntary running exercise alters microbiota composition and increases n-butyrate concentration in the rat cecum. *Bioscience, Biotechnology, and Biochemistry*.

[52] Perrin P., Pierre F., Patry Y. (2001). Only fibres promoting a stable butyrate producing colonic ecosystem decrease the rate of aberrant crypt foci in rats. *Gut*.

[53] Evans C. C., LePard K. J., Kwak J. W. (2014). Exercise prevents weight gain and alters the gut microbiota in a mouse model of high fat diet-induced obesity. *PLoS ONE*.

[54] Queipo-Ortuño M. I., Seoane L. M., Murri M. (2013). Gut microbiota composition in male rat models under different nutritional status and physical activity and its association with serum leptin and ghrelin levels. *PLoS ONE*.

[55] Stilling R. M., Ryan F. J., Hoban A. E. (2015). Microbes & neurodevelopment—absence of microbiota during early life increases activity-related transcriptional pathways in the amygdala. *Brain, Behavior, and Immunity*.

[56] Diaz Heijtz R., Wang S., Anuar F. (2011). Normal gut microbiota modulates brain development and behavior. *Proceedings of the National Academy of Sciences of the United States of America*.

[57] Marra L., Cantile M., Scognamiglio G. (2013). Deregulation of HOX B13 expression in urinary bladder cancer progression. *Current Medicinal Chemistry*.

[58] Bravo J. A., Forsythe P., Chew M. V. (2011). Ingestion of Lactobacillus strain regulates emotional behavior and central GABA receptor expression in a mouse via the vagus nerve. *Proceedings of the National Academy of Sciences of the United States of America*.

[59] Forsythe P., Bienenstock J., Kunze W. A. (2014). Vagal pathways for microbiome-brain-gut axis communication. *Advances in Experimental Medicine and Biology*.

[60] Cook M. D., Allen J. M., Pence B. D. (2016). Exercise and gut immune function: evidence of alterations in colon immune cell homeostasis and microbiome characteristics with exercise training. *Immunology and Cell Biology*.

[61] Allen J. M., Miller M. E. B., Pence B. D. (2015). Voluntary and forced exercise differentially alters the gut microbiome in C57BL/6J Mice. *Journal of Applied Physiology*.

[62] Petriz B. A., Castro A. P., Almeida J. A. (2014). Exercise induction of gut microbiota modifications in obese, non-obese and hypertensive rats. *BMC Genomics*.

[63] Woo J., Shin K., Park S., Jang K., Kang S. (2013). Effects of exercise and diet change on cognition function and synaptic plasticity in high fat diet induced obese rats. *Lipids in Health and Disease*.

[64] Chieffi S., Conson M., Carlomagno S. (2004). Movement velocity effects on kinaesthetic localisation of spatial positions. *Experimental Brain Research*.

[65] Molteni R., Wu A., Vaynman S., Ying Z., Barnard R., Gómez-Pinilla F. (2004). Exercise reverses the harmful effects of consumption of a high-fat diet on synaptic and behavioral plasticity associated to the action of brain-derived neurotrophic factor. *Neuroscience*.

[66] Chieffi S., Iavarone A., Viggiano A., Monda M., Carlomagno S. (2012). Effect of a visual distractor on line bisection. *Experimental Brain Research*.

[67] Li W., Dowd S. E., Scurlock B., Acosta-Martinez V., Lyte M. (2009). Memory and learning behavior in mice is temporally associated with diet-induced alterations in gut bacteria. *Physiology and Behavior*.

[68] Chieffi S., Iachini T., Iavarone A., Messina G., Viggiano A., Monda M. (2014). Flanker interference effects in a line bisection task. *Experimental Brain Research*.

[69] Kang S. S., Jeraldo P. R., Kurti A. (2014). Diet and exercise orthogonally alter the gut microbiome and reveal independent associations with anxiety and cognition. *Molecular Neurodegeneration*.

[70] Klaenhammer T., Altermann E., Arigoni F. (2002). Discovering lactic acid bacteria by genomics. *Lactic Acid Bacteria: Genetics, Metabolism and Applications*.

[71] Estaki M., Pither J., Baumeister P. (2016). Cardiorespiratory fitness as a predictor of intestinal microbial diversity and distinct metagenomic functions. *The FASEB Journal*.

[72] Claesson M. J., Jeffery I. B., Conde S. (2016). Gut microbiota composition correlates with diet and health in the elderly. *Nature*.

[73] Monda M., Messina G., Scognamiglio I. (2014). Short-term diet and moderate exercise in young overweight men modulate cardiocyte and hepatocarcinoma survival by oxidative stress. *Oxidative Medicine and Cellular Longevity*.

[74] Handschin C., Spiegelman B. M. (2008). The role of exercise and PGC1*α* in inflammation and chronic disease. *Nature*.

[75] Franceschi C., Campisi J. (2014). Chronic inflammation (inflammaging) and its potential contribution to age-associated diseases. *The Journals of Gerontology Series A: Biological Sciences and Medical Sciences*.

[76] Chieffi S., Secchi C., Gentilucci M. (2009). Deictic word and gesture production: their interaction. *Behavioural Brain Research*.

[77] Everard A., Belzer C., Geurts L. (2013). Cross-talk between Akkermansia muciniphila and intestinal epithelium controls diet-induced obesity. *Proceedings of the National Academy of Sciences*.

[78] Juneau M., Hayami D., Gayda M., Lacroix S., Nigam A. (2014). Provocative issues in heart disease prevention. *Canadian Journal of Cardiology*.

[79] Vgontzas A. N. (2008). Does obesity play a major role in the pathogenesis of sleep apnoea and its associated manifestations via inflammation, visceral adiposity, and insulin resistance?. *Archives of Physiology and Biochemistry*.

[80] Tan X., Saarinen A., Mikkola T. M. (2013). Effects of exercise and diet interventions on obesity-related sleep disorders in men: study protocol for a randomized controlled trial. *Trials*.

[81] Morris G., Berk M., Galecki P., Walder K., Maes M. (2016). The neuro-immune pathophysiology of central and peripheral fatigue in systemic immune-inflammatory and neuro-immune diseases. *Molecular Neurobiology*.

[82] Maes M. (2009). Inflammatory and oxidative and nitrosative stress pathways underpinning chronic fatigue, somatization and psychosomatic symptoms. *Current Opinion in Psychiatry*.

[83] Morris G., Maes M. (2014). Oxidative and nitrosative stress and immune-inflammatory pathways in patients with myalgic encephalomyelitis (ME)/chronic fatigue syndrome (CFS). *Current Neuropharmacology*.

[84] Frémont M., Coomans D., Massart S., De Meirleir K. (2013). High-throughput 16S rRNA gene sequencing reveals alterations of intestinal microbiota in myalgic encephalomyelitis/chronic fatigue syndrome patients. *Anaerobe*.

[85] Chen Y.-M., Wei L., Chiu Y.-S. (2016). *Lactobacillus plantarum* TWK10 supplementation improves exercise performance and increases muscle mass in mice. *Nutrients*.

[86] Duncan S. H., Louis P., Flint H. J. (2004). Lactate-utilizing bacteria, isolated from human feces, that produce butyrate as a major fermentation product. *Applied and Environmental Microbiology*.

